# Modelling of Asphalt's Adhesive Behaviour Using Classification and Regression Tree (CART) Analysis

**DOI:** 10.1155/2019/3183050

**Published:** 2019-08-15

**Authors:** Md Arifuzzaman, Uneb Gazder, Md Shah Alam, Okan Sirin, Abdullah Al Mamun

**Affiliations:** ^1^Department of Civil Engineering, University of Bahrain, Zallaq, Bahrain; ^2^Department of Civil and Architectural Engineering, Qatar University, Doha, Qatar

## Abstract

The modification by polymers and nanomaterials can significantly improve different properties of asphalt. However, during the service life, the oxidation affects the constituents of modified asphalt and subsequently results in deviation from the desired properties. One of the important properties affected due to oxidation is the adhesive properties of modified asphalt. In this study, the adhesive properties of asphalt modified with the polymers (styrene-butadiene-styrene and styrene-butadiene) and carbon nanotubes were investigated. Asphalt samples were aged in the laboratory by simulating the field conditions, and then adhesive properties were evaluated by different tips of atomic force microscopy (AFM) following the existing functional group in asphalt. Finally, a predictive modelling and machine learning technique called the classification and regression tree (CART) was used to predict the adhesive properties of modified asphalt subjected to oxidation. The parameters that affect the behaviour of asphalt have been used to predict the results using the CART. The results obtained from CART analysis were also compared with those from the regression model. It was observed that the CART analysis shows more explanatory relationships between different variables. The model can predict accurately the adhesive properties of modified asphalts considering the real field oxidation and chemistry of asphalt at a nanoscale.

## 1. Introduction

The researchers usually modify the asphalt using different types of polymers to provide more durable and sustainable pavements. The polymers used for the modification of asphalt tend to have large chains (straight or cross-linked). The chemistry and structure of the chains affect the behaviour of the polymer as well as polymer-modified asphalt (PMA). The most commonly used polymers are elastomers and plastomers. The elastomers improve the elastic properties, while the plastomers provide a plastic matrix in the modified asphalt. In the following sections, the PMA indicates only styrene-butadiene-styrene (SBS) and styrene-butadiene (SB) modified asphalt following the scope of this study.

SBS is one of the most widely used polymers in the asphalt industry, followed by reclaimed tire rubber [1], which improves the mechanical, physical, and rheological properties of asphalt mixtures [2], increases the elasticity and tensile properties of asphalt [3], and lowers the creep stiffness [4]. The SB product is an SBS block copolymer and elastomeric in nature. The use of SB can affect different properties of asphalt, including viscoelastic properties (Jnr) [5], and provide increased resistance to permanent deformation at moderate temperature (25°C) [6], low-temperature ductility [7], etc. In addition to providing different aforementioned properties, it is observed that the use of SB and SBS significantly affects the adhesive properties of asphalt [7]. However, the effectiveness of the improved properties, including the adhesive properties, changes gradually over the service life. There could be different factors that affect the desired properties of PMA, and oxidation of asphalt is one of them. Oxidation can alter the constituents, as well as the adhesion of the asphalt that subsequently erodes the viscoelastic properties and results in asphalt pavement failure [8, 9]. Therefore, it is imperative to have an explanatory insight to predict the adhesive property of the oxidized PMA.

In addition to the use of polymer, the increased traffic, the desired improved properties of the asphalt pavement, and the rapid development of nanotechnology have led the researchers to focus on introducing nanomaterials for asphalt modification. Nanomaterials are described as having at least one dimension within 1–100 nm. The properties of nanosized particles differ from those of traditional materials because of the increased ratio of surface to volume and nanometer-sized plates [10]. It was also observed that nanomaterials showed high sensitivity to temperature, high ductility, high surface area, high tension resistance, low electrical resistance, etc. [11–15]. Because of these favourable properties, a large number of nanomaterials have been used for asphalt modification, and carbon nanotube (CNT) is one of them. The CNT was found to improve the tracking resistance and thermal cracking [16, 17]. It can significantly improve the rheological and adhesive properties of asphalt, and the increase in the content of CNTs results in highly viscous and elastic coefficient values regardless of the type of binder [18–20]. However, it is observed that the presence of nanomaterials (or filler materials) affects the adhesive properties of asphalt [21]. In addition to this, the oxidation can also affect the adhesive properties of CNT-modified asphalt (CMA) [22]. Therefore, it is also important to visualize and predict the changes in adhesive properties of oxidized CMA.

Based on the above discussion, it is revealed that the adhesive properties play a significant role for oxidized asphalt regardless of the type of modified asphalt (PMA/CMA). However, none of the previous studies attempted to predict the adhesive properties of oxidized asphalt to the best of authors' knowledge. Some of the studies that addressed the adhesive properties of the PMA [23–25] or CMA [26, 27] considered the effect of moisture rather than oxidization. In this regard, this study predicted the adhesive properties of oxidized asphalt (modified by polymers and CNTs) using a predictive modelling and machine learning technique, i.e., the classification and regression tree (CART). The model addresses the adhesive properties of modified asphalt simulating the real field oxidation and chemistry of asphalt at a nanoscale.

## 2. Research Approach

**Figure 1:**
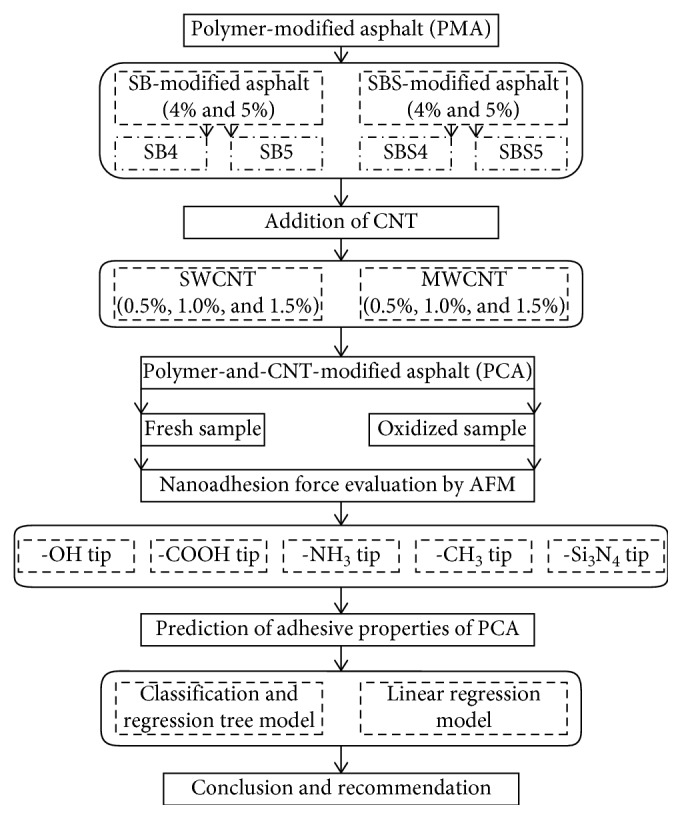
Flow chart of the analysis steps.

The flow chart describing the analysis steps involved in this study can be seen in Figure 1. It can be seen from the figure that the PMA will be modified using two different types of CNTs (each one comprises three different percentages). Once the PMA is modified by a CNT (named PCA), the samples are divided into two groups, such as fresh and oxidized. The adhesive properties of each sample are analysed using five different tips of AFM. The parameters (percentage and type of CNT, functional group, polymer type, etc.) that affect the behaviour of asphalt have been used to predict adhesive properties using the CART and were compared with the regression model. Further details can be observed in the following sections.

## 3. Materials and Procedure

The base asphalt collected from a local distributor was evaluated in the laboratory, and its properties are given in Table 1. The base asphalt was modified using 4% and 5% of SB and SBS following the usual practice in the industry [28]. Each polymer-modified asphalt was further modified using two different types of CNTs. The CNT is a one-atom thick graphite plate made into a seamless, one-nanometer diameter hollow cylinder. The synthesis and characterization of the helical microtubules of the fibre are performed on a molecular scale of the structures. The CNT exists in the form of coaxial tubes (multiwalled CNTs) and single tubes (single-walled CNTs). Young's modulus of a CNT, depending on the radius of the tube, can be up to 1,000 GPa, and the tensile strength can be up to 150 GPa [29].

### 3.1. AFM Testing Description

**Figure 2:**
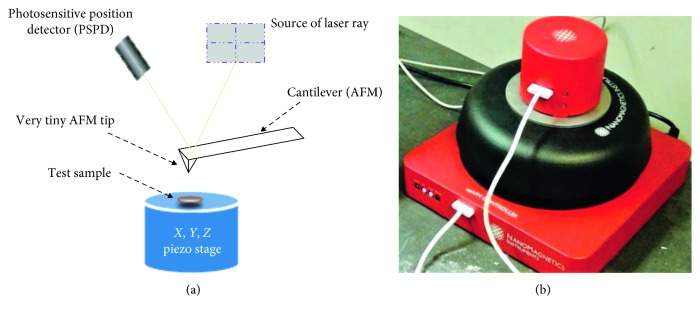
(a) Schematic of AFM. (b) AFM machine used in this study.

Atomic force microscopy (AFM) can be a very important and suitable tool to assess the nanomechanical properties of asphalt such as contact force, friction, and van der Waals force. Several studies attempted to study different properties of asphalt using the AFM [30–33]. The AFM has also been used to evaluate different nanomechanical properties including adhesion and cohesion of asphalt binders [34]. Some of the studies observed the changes in adhesion of asphalt due to the presence of SBS [35], antistripping agents [36], CNTs [37], etc. In any AFM testing setup, the outer surface of the asphalt binder is simply probed with a sharp and tiny tip. The tip is positioned at the end of the cantilever. The existing attractive/repulsive force between the tip and sample makes the cantilever to deflect or bend. A photosensitive position detector (PSPD) with a built-in laser beam reflection system measures the bending of the cantilever. The deflection of the cantilever is then multiplied with its spring constant to find the acting attractive/repulsive force between the tip and sample surface according to the following equation:

(1) $$\begin{array}{c} F=-cd, \end{array}$$

where *F*, *c*, and *d* are the force, spring constant, and vertical displacement, respectively. A simplified schematic diagram of AFM is shown in Figures 2(a), and 2(b) presents the diagram of the AFM machine used in this study. All the experiments are conducted in a clean room to be safe from different pollutants in the air (like chemical vapours, aerosol, dust, and airborne microbes).

#### 3.1.1. Tip Functionalization

In this study, the original AFM tips were made of a silicon nitrite (-Si_3_N_4_) material which was later on modified with ammine (-NH_3_), hydroxy (-OH), methyl (-CH_3_), and carboxyl (-COOH) functional groups. It was carried out by probing an asphalt film surface modified with polymers with a functionalized AFM tip that facilitates the measurement of the intermolecular forces between two asphalt molecules.

The tip functionalization procedure follows a precise deposition of a thin, monolayer film on the tip. The immersion of the AFM tip was carried out using chlorosilane solution or organic thiol [38].

### 3.2. Sample Preparation

The fresh asphalt sample was heated inside a laboratory container at a temperature of around 164°C. After 30–45 minutes of duration, the asphalt binder was mixed with the polymer fraction chosen, as well as the CNT. The mixed samples modified with the CNT and polymer are called dry conditioned samples. The samples were placed on a glass substrate having a dimension of approximately 10 mm × 10 mm × 1 mm. The dry samples were placed inside a draft oven with an elevated temperature of 60°C for seven days that simulates the anticipated aging in the field condition.

## 4. Classification and Regression Tree (CART) Analysis

The multiple regression model and the classification and regression tree (CART) approaches were used to understand the effect of different variables on the adhesion force of asphalt. The use of regression models requires an assumption regarding the underlying distribution of the data, and it is a parametric method. On the contrary, the nonparametric technique like artificial neural networks (ANNs) has also been used that lacks the explanatory capability. The CART is a nonparametric technique that can be used to include variable(s) at more than one stage of the tree. Therefore, complex interdependencies can also be uncovered among the variables. The CART has successfully handled the complex nonlinearity between the predictors and response with its adaptive interpretation skills [39]. It can handle the multicollinearity problems of the data more appropriately compared to the regression models. In addition to this, the CART analysis provides a model that can be interpreted through logical statements to understand the effect of different variables on the target variable that is often not found in other data mining tools [40]. The application of the CART was successfully used not only to understand and predict consumers' behaviour but also in the road safety research (i.e., car seat belt use). It was also used in different sectors of pavement engineering, such as evaluation of the field serviceability of pothole patches [41], factors influencing permeability of the rigid pavement [42], and roughness of the asphalt pavement [43], field prediction of maintenance probability, and selection of certain maintenance approaches following the existing condition [44, 45]. However, the studies in which predictive models are used for predicting adhesion force of asphalt were found to be very few, and the use of the CART technique has not been found in these studies. Therefore, this technique has been applied for predictive modelling of oxidized asphalt for the first time. The details of the variables used in the model are presented in Table 2.

## 5. Results and Discussion

The CART analysis incorporated 240 samples where one hundred sixty samples were used for training and eighty samples were used for testing.  Table 3 presents the accuracies for training and test samples in CART analysis.

The accuracy was calculated via the coefficient of correlation, root mean square error (RMSE), and mean absolute percentage error (MAPE) for samples used for the training and testing of the model. These values were calculated by using the actual/target values from the lab test and model predictions. It can be observed from the table that the coefficient of correlation (CC) was reasonable for training as well as test samples. The error was approximately 27% (mean absolute percentage error (MAPE)) for the test samples which amounts to 53 kN (root mean square error (RMSE)) in terms of adhesion force. The accuracy measures of the CART were found to be acceptable for training as well as test samples. However, there was no drastic change in the error values which indicates that the model did not overfit the training samples. Different artificial intelligence (AI) techniques, such as multilayer perceptions (MLPs), support vector machines (SVMs), and adaptive network fuzzy inference systems (ANFISs), have been used for predicting adhesion force for asphalt [46]. However, for pavement design, these techniques cannot explain the relationship between the variables considered in the study, which makes the use of the proposed model in decision-making difficult. This gap can be fulfilled by the CART which explains the relationships between variables.

**Figure 3:**
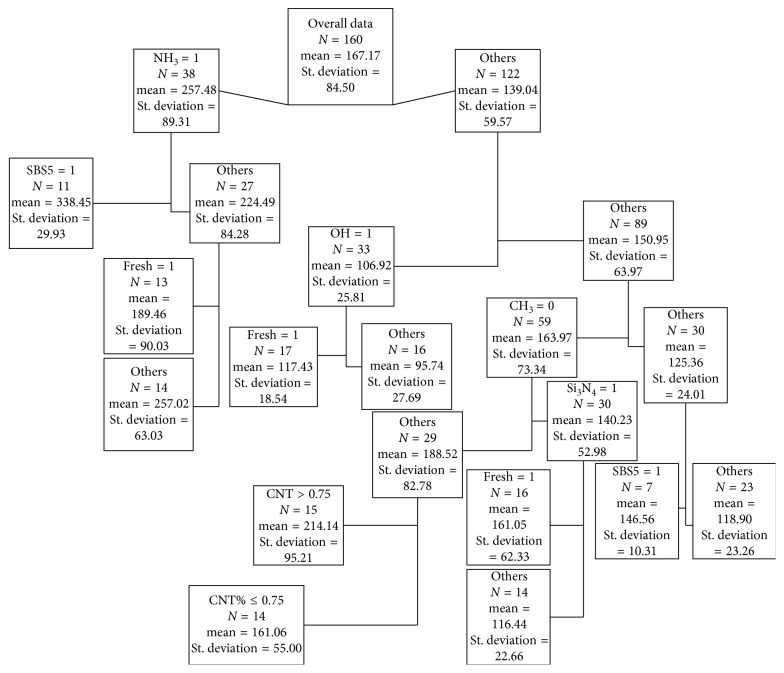
CART for predicting asphalt adhesion force.

The CART presented in Figure 3 highlights the following points. The most important parameter was found to be the tip type, i.e., NH_3_, which is at the top of the tree. The use of NH_3_ in the tip increases the adhesion force of asphalt. Hence, AFM tests need to be designed properly before using their results for mix design. The highest adhesion force was found when the NH_3_ tip was used and SBS5 was used as the binder as this node has the highest mean adhesion force. The SBS5 binder was also found to increase the adhesion force when used with other tip types except CH_3_. Therefore, it could be said that having dual styrene bonds increases the adhesion force of asphalt which can be attributed to a higher degree of internal bonding for the additive. Nodes for fresh samples were found to have a higher adhesion force as expected. The lowest mean adhesion force was observed for aged samples when the tip was made of OH. The CNT type was not found to have any effect on the adhesion force. However, it may have impact on other properties of asphalt such as elasticity, viscosity, and density. Hence, further research is required with regard to this modification in asphalt. The CART model provides the mean adhesion force for each combination of variables, and hence, it can be directly used to develop guidelines for mix design of asphalt and design of AFM experiments for asphalt. Equation (2) represents the regression model developed for this study. It was developed by using the method of ordinary least squares. The coefficients were checked for their statistical significance, and the variables with statistically insignificant coefficients were omitted. The accuracy of this model is given in Table 4.

(2) $$\begin{array}{c} \text{AF}=188.52-48.28\left({\text{Si}}_{3}{\text{N}}_{4}\right)+68.96\left({\text{NH}}_{3}\right)-81.6\left(\text{OH}\right)-63.16\left({\text{CH}}_{3}\right). \end{array}$$

The following point was observed while comparing the regression model with the CART: the accuracy of the CART model (Table 3) is higher for training and test samples than that of the regression model (Table 4).

The tip type NH_3_ was found to have the highest positive impact, and OH had the highest negative impact on the regression model (see equation (2)). Similar observations can also be observed from the CART. The correlation of the tip type, binder type, and freshness of the samples and their combined impact on adhesion force are not captured by the regression model but were clearly shown in the CART.

## 6. Conclusions and Recommendations

This study investigated the aging behaviour of asphalts modified with SB, SBS, and CNTs. The aging behaviour is measured by evaluating the changes in adhesive properties of modified asphalt (SB, SBS, and CNTs) at the nanoscale. The test results were predicted by using classification and regression tree (CART) analysis including different parameters that affect the aging behaviour of modified asphalts. CART results were compared with the regression model results. The main findings from this study can be summarized as follows:

1. The CART analysis shows more explanatory relationships, at different levels of the tree, between different variables that affect the behaviour of oxidized asphalt.
2. The CART results were found to be more accurate (with higher CC and lower MAPE and RMSE values) than those of the regression model. It could be due to consideration of the interaction effect in the CART model that differs significantly from the usual regression techniques [47].
3. The functional group -NH_3_ was the most important parameter for the tip type. The use of -NH_3_ in the tip increases the adhesion force of asphalt. Hence, this effect should be considered when designing the AFM experiments for asphalt adhesion to avoid any biasness of the results due to the type of tip.
4. The highest adhesion force was found when the -NH_3_ tip was used with the SBS5 binder as this node has the highest mean adhesion force, whereas the lowest mean adhesion force was observed for aged samples when the tip was made of -OH.
5. The CNT type was not found to have any effect on the adhesion force.
6. In addition to this, scrutinizing the relation between the nanoscale adhesion and different macrostructural changes can provide a rigorous conclusion for hot mix asphalt (HMA).

## Figures and Tables

**Table 1:** Asphalt properties.

Properties	Values
Specific gravity	1.02
Viscosity (centipoise)	500
Performance grade	66-22

**Table 2:** Variables used in the model.

Variable	Explanation
Fresh	Binary variable: 1 for fresh samples or otherwise 0
Si_3_N_4_, NH_3_, OH, CH_3_	Binary variables for tip type: 1 for a specific tip type or otherwise 0. Tip type is 0 for COOH
SB4, SB5, SBS4, SBS5	Binary variables for binder type: 1 for a specific binder type or otherwise 0
CNT%	% of carbon nanotubes
SWNT (or CNT)	Binary variable for CNT type: 1 for SWNT type and 0 for MWNT type

**Table 3:** Accuracy measures of the CART.

Accuracy measure	Training sample	Test sample
CC	0.77	0.64
RMSE	46.10	53.02
MAPE	23.42%	26.82%

**Table 4:** Accuracy measures of the linear regression model.

Accuracy measure	Training sample	Test sample
CC	0.67	0.64
RMSE	44.81	62.52
MAPE	24.29%	32.95%

## Data Availability

The data are available from the corresponding author upon request.
